# Do Digital Handover Checklists Influence the Clinical Outcome Parameters of Intensive Care Unit Patients? A Randomized Controlled Pilot Study

**DOI:** 10.3389/fmed.2021.661343

**Published:** 2021-04-20

**Authors:** Nina Verholen, Lina Vogt, Martin Klasen, Michelle Schmidt, Stefan Beckers, Gernot Marx, Saša Sopka

**Affiliations:** ^1^Department of Anaesthesiology, Medical Faculty, University Hospital Rheinisch-Westfälische Technische Hochschule Aachen, Aachen, Germany; ^2^AIXTRA—Competence Center for Training and Patient Safety, Medical Faculty, Rheinisch-Westfälische Technische Hochschule Aachen, Aachen, Germany; ^3^Department of Intensive Care Medicine and Intermediate Care, Medical Faculty, University Hospital Rheinisch-Westfälische Technische Hochschule Aachen, Aachen, Germany

**Keywords:** standardized handover, checklists, ISBAR_3_, ICU, patient safety, study design, pilot study, feasibility

## Abstract

**Background:** Clinical handovers have been identified as high-risk situations for medical treatment errors. It has been shown that handover checklists lead to a reduced rate of medical errors and mortality. However, the influence of handover checklists on essential patient outcomes such as prevalence of sepsis, mortality, and length of hospitalization has not yet been investigated in a randomized controlled trial (RCT).

**Objectives:** The aim of the present pilot study was to estimate the effect of two different handover checklists on the 48 h sepsis-related organ failure assessment (SOFA) score and the feasibility of a respective clinical RCT.

**Methods:** Outcome parameters and feasibility were investigated implementing and comparing an intervention with a control checklist.

**Design:** Single center two-armed cluster randomized prospective crossover pilot study.

**Setting:** The study took place over three 1-month periods in an intensive care unit (ICU) setting at the University Hospital Aachen.

**Patients/Participants:** Data from 1,882 patients on seven ICU wards were assessed, of which 1,038 were included in the analysis.

**Intervention:** A digital standardized handover checklist (ISBAR_3_) was compared to a control checklist (VICUR).

**Main Outcome Measures:** Primary outcome was the 2nd 24 h time window sepsis-related organ failure assessment (SOFA) score. Secondary outcomes were SOFA scores on the 3rd and 5th 24 h time window, mortality, reuptake, and length of stay; handover duration, degree of satisfaction, and compliance as feasibility-related outcomes.

**Results:** Different sepsis scores were observed only for the 1st 24 h time window after admission to the ICU, with higher values for ISBAR_3_. With respect to the patient-centered outcomes, both checklists achieved similar results. Average handover duration was shorter for VICUR, whereas satisfaction and compliance were higher for ISBAR_3_. However, overall compliance was low (25.4% for ISBAR_3_ and 15.8% for VICUR).

**Conclusions:** Based on the results, a stratified randomization procedure is recommended for following RCTs, in which medical treatment errors should also be investigated as an additional variable. The use of control checklists is discouraged due to lower acceptance and compliance among healthcare practitioners. Measures should be undertaken to increase compliance with the use of checklists. Clinical outcome parameters should be carefully selected.

**Trial Registration:** ClinicalTrials.gov, Identifier [NCT03117088]. Registered April 14, 2017.

## Introduction

Improving safety for patients in health care is a crucial, yet challenging endeavor. The World Health Organization (WHO) defines patient safety as “the absence of preventable harm to a patient during the process of health care and reduction of risk of unnecessary harm associated with health care to an acceptable minimum” (1). High prevalence rates of procedural errors in health care, often leading to patient harm, emphasize the relevance of the patient safety concept. As an example, the WHO points out that medical errors and health care related events occur in 8–12% of hospitalized patients in Europe (2). Fifty to seventy percent of these medical errors could be avoided by comprehensive systematic strategies (2), such as evidence-based interventions (3, 4). Accordingly, patient safety has increasingly gained public (4, 5) and scientific interest (6) and a growing awareness of the importance of patient safety in clinical practice.

In the course of continuous medical and technological development and the increasing complexity of health care, error possibilities rise. During treatment, patients are cared for in an interdisciplinary and interprofessional setting. Moreover, the treatment often takes place in different locations and the treatment team varies frequently due to shift changes. This complexity creates interface situations such as clinical handovers, which have been identified as high-risk situations for medical treatment errors (7–10).

Being a long-identified risk factor in other safety-related domains such as aviation (11), inadequate communication is a threat to patient safety as well (10, 12). A largescale European Commission project considers deficient handover communication as the cause of 25–40% of all adverse events (13). Similarly, according to McSweeney et al. (14) communication failures during handover lead to negative effects on patient care, such as subsequent medication errors, inaccurate patient plans or delayed hospital discharges. Additionally, Starmer et al. (15) substantiated the relationship between poor handovers, errors, and preventable adverse events.

The identification of handovers as risk situations (16) has made them a main target of patient safety initiatives (17, 18). Handovers regarding intensive care patients may be especially critical as these patients are often unable to verbalize their own health care problems and needs. Accordingly, the Agency for Healthcare Research and Quality (AHRQ) and the Accreditation Council for Graduate Medical Education (ACGME) have declared the improvement of handovers as a top priority of the US-wide efforts to improve patient safety (15). To increase patient safety in handover situations, the use of checklists has been recommended (19, 20). Checklists support memory and attention and standardize the communication in handover situations. Thus, they can help to prevent misunderstandings and the loss of valuable information. It has been shown that the implementation of checklists leads to a reduced rate of medical errors (15). However, research has only recently begun to address the impact of handover checklists on clinical patient outcomes (21). So far, no clinical trials have investigated the influence of handover checklists on essential parameters such as patient mortality, prevalence of sepsis, and length of hospitalization in a randomized controlled trial.

The aim of the present pilot study was to estimate the effect size of a checklist intervention on the emergence of sepsis after 48 h for a future randomized controlled trial (RCT). Moreover, a second aim was to evaluate feasibility aspects of the respective RCT.

**Figure 1:**
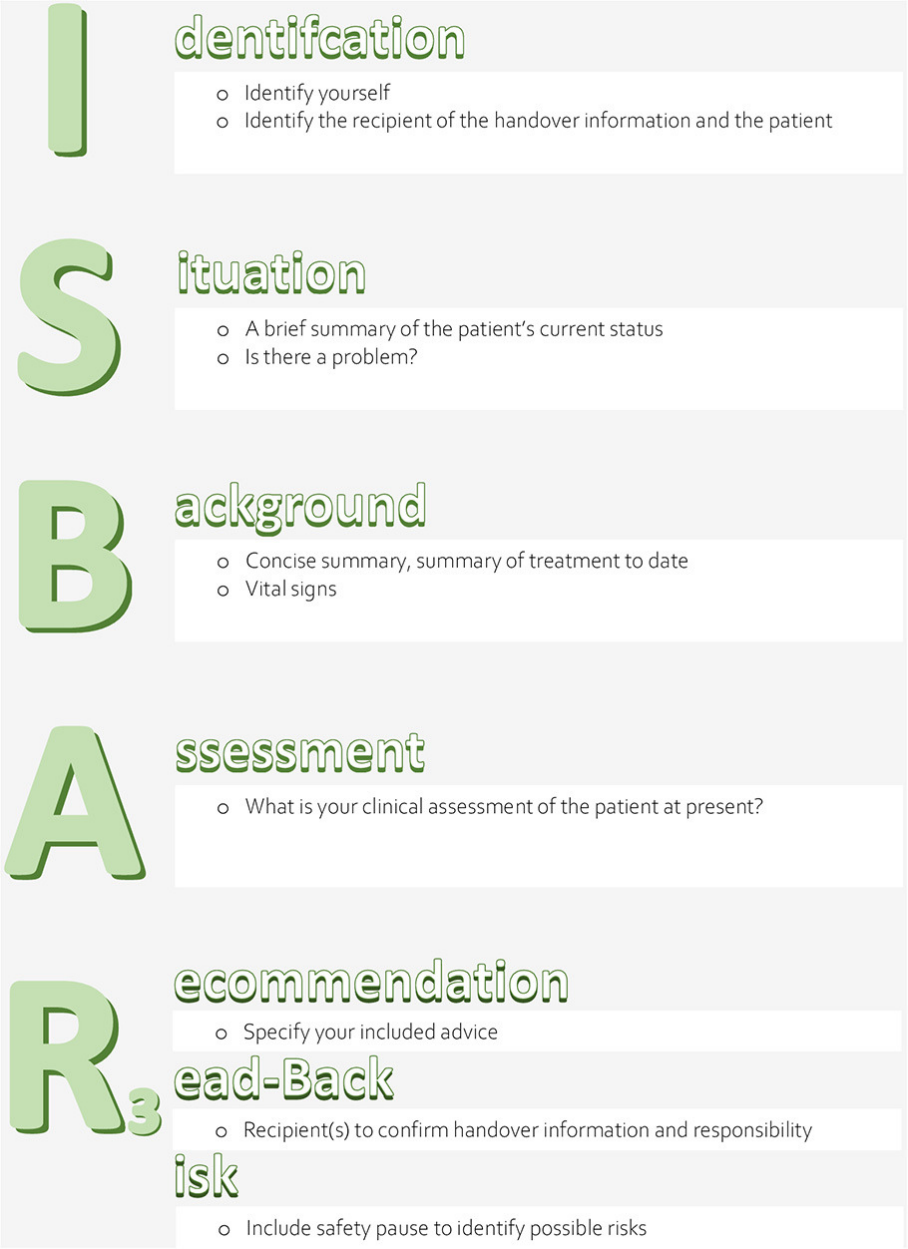
The ISBAR_3_ checklist.

**Figure 2:**
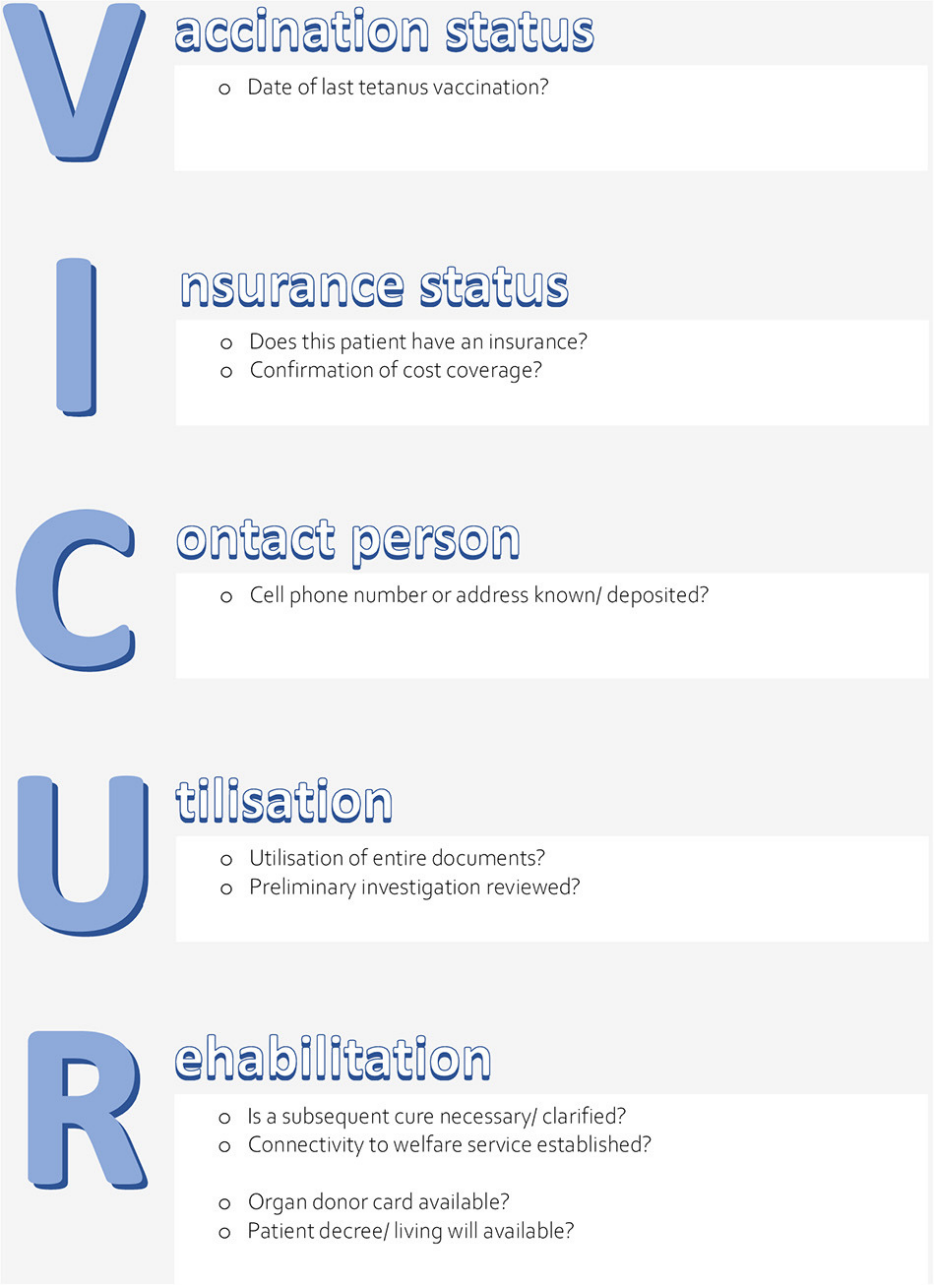
The VICUR checklist.

For this purpose, the present pilot study evaluated the following aspects:

1. The potential effect of a structured clinical handover checklist (ISBAR_3_: Identification, Situation, Background, Assessment, Recommendation, Read-back, Risk) (see Figure 1) on patient-related objective outcome parameters (sepsis-related organ failure assessment (SOFA) scores after 48 h (primary outcome) and on mortality, reuptake, and length of stay (secondary outcomes) in an ICU setting. To exclude a potential bias of unspecific observer effects (22), a control condition was employed, i.e., an unspecific handover checklist (VICUR: vaccination status, insurance status, contact person, utilization, rehabilitation) (see Figure 2).
2. As feasibility-related outcome parameters, we evaluated handover duration, degree of satisfaction, and compliance among the ICU staff. The aim of the feasibility aspect was to evaluate a large-scale dummy run of the trial procedures and to determine recruitment and compliance rates; moreover, our aims were to estimate the effect size for our primary outcome parameter and to evaluate the suitability of the primary and secondary outcome parameters for a future large-scale RCT (23).

## Materials and Methods

### Ethics Approval

This study was performed in line with the principles of the Declaration of Helsinki. The study protocol was approved by the Ethical Committee of the Faculty of Medicine of RWTH Aachen University (Chairperson Prof. Dr. med. G. Schmalzing) (EK 075/17) on May 24th, 2017.

### Study Setting

The single center pilot study was conducted during 6 months (May–October 2017) simultaneously on seven ICU wards at the University Hospital RWTH Aachen, Germany (Department of Intensive Care Medicine and Intermediate Care), providing 105 operative intensive care beds and focusing in part on a specific patient clientele. The main focal points are neurosurgery, cardiac surgery, visceral surgery, burn surgery, and weaning. Surgical ICU patients outside of this scope can be admitted to any of the ICU wards.

### Participants, Inclusion, and Exclusion Criteria

All physicians working on ICU wards during data acquisition received the study information and signed the written consent. They were informed that their participation was voluntary and could be discontinued at any time without explanation or any disadvantages. The medical personnel was informed how to use the tablet PC, but there was no special training on how to use the checklist to minimize the risk of performance bias. Those meeting the following criteria were excluded: chief of the department, colleagues involved in study group or expert group.

All patients treated on the ICU wards during the study were included in the analysis except for patients with less than two documented handovers with the checklist. Moreover, all patients under the age of 18 years and pregnant women were excluded due to different standard values for the patient-related outcomes.

### Study Design

**Figure 3:**
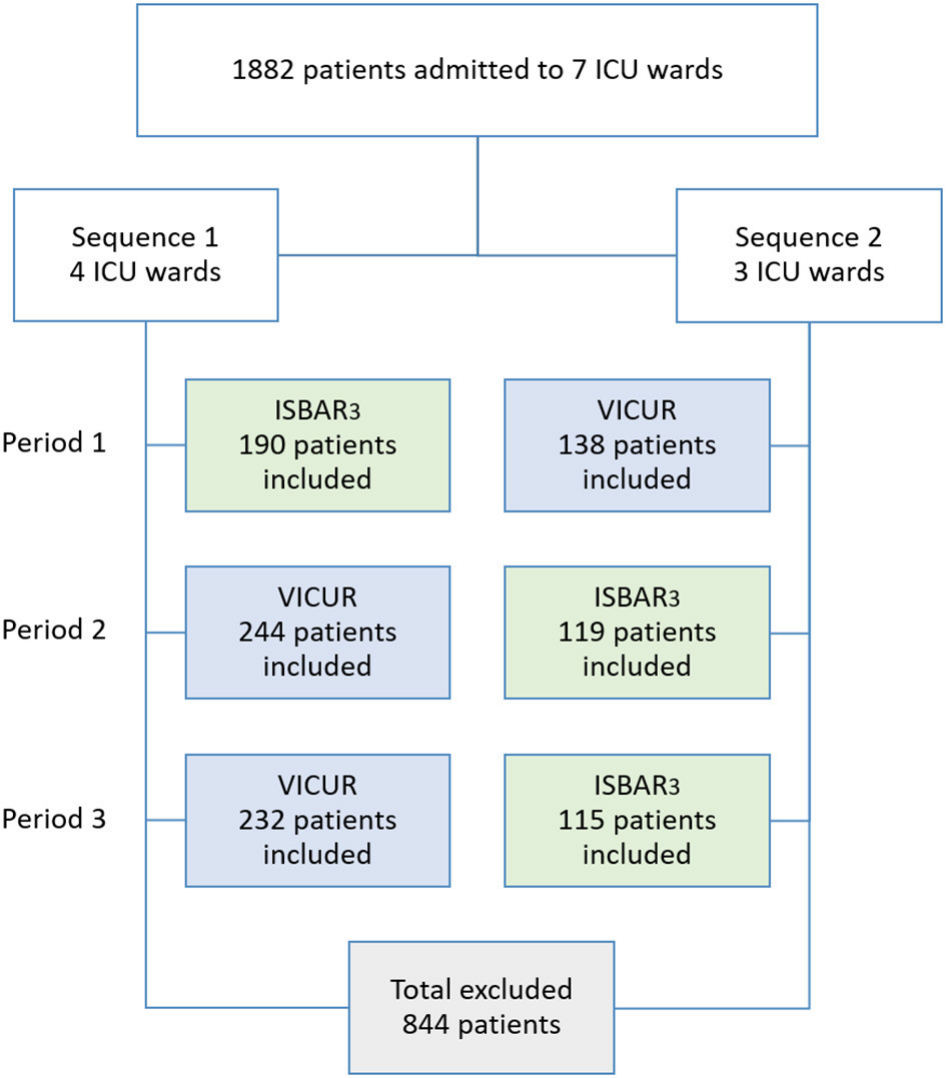
Study design.

We conducted a two-armed cluster-randomized crossover prospective single center pilot study, using a three-period two-conditions layout, in which ISBAR_3_ (A) and VICUR (B) served as two conditions. Specifically, we used a balanced model with the two sequences ABB and BAA. The participating ICU wards were assigned to two sequences by a random allocation rule with a ratio of 3:4 (Figure 3). Randomization lists were created with randomizeR (24) and conducted by our statistician.

Three times a data collection period of one month was followed by a one-month wash-out period (30 days each). During the data collection periods, two different checklists were used (see Intervention section for details) and online satisfaction questionnaires were delivered afterwards. Blinding was reached by concealing the purpose of both checklists from the study participants, i.e., the physicians were not told what the idea behind the ISBAR_3_ and VICUR checklists was. Respectively participants were instructed to carry out the bedside handover as usual—performing it at the physicians' shift changes every 12 h—while using the checklists and ticking off all items. The participants did not know in advance which checklist would be used.

### Intervention

An *online based application* (app), to which the physicians had access via tablets (iPad Mini®), was created in collaboration with the Department for Medical Statistics. The use of the app had the following benefits: Recording the demographic data (e.g., age, function, or experience) of the performing physicians, providing the checklists, and checking off of items that have already been completed, recording of time and assignment of the recorded data to the patient. Brief instructions for the usage of the tablets and the study-procedure were performed before data collection.

Physicians were instructed to use the checklists as mnemonic and structuring aid during shift-to-shift handovers that took place twice a day. The two *checklists* used were ISBAR_3_ and VICUR.

*Checklist ISBAR*_3_ (Figure 1): The concept SBAR (Situation, Background, Assessment, Recommendation), initially established in the US Navy (16), is a communication tool that creates the conditions for an effective, succinct, timely, and consistent transfer of communication (16) in complex situations (25). It is standardized, simple, structured, flexibly applicable to a wide variety of settings (26) and has also been adapted to healthcare (27, 28). The use of SBAR allows the reduction or avoidance of errors caused by misunderstandings, loss of information, or misinterpretation (26). It has been recommended by the WHO (29), the Joint Commission (30), and the German Society of Anaesthesiology and Intensive Care Medicine (DGAI) (31) for use during handover as well as by the Institute for Healthcare Improvement (IHI) (32) and the Agency for Healthcare Research and Quality (AHRQ) (33) for use in critical situations.

ISBAR_3_ (Identification, Situation, Background, Assessment, Recommendation, Read-back, Risk) is an adaptation of SBAR. The addition of the letter “I” is intended to ensure knowledge of the identity of the conversation partners and the patient they are talking about (7) and to guarantee mutual attention. Adding two “Rs” allows the sender to check if the conversation partner has received the transmitted information (7, 34) and enables possible further inquiries (10). Potential risks for the subsequent patient treatment are pointed out.

*Checklist VICUR* (Figure 2): VICUR (Vaccination status, Insurance status, Contact person, Utilization, Rehabilitation) is a checklist developed by a group of experts inspired by the “Project White List” (“Projekt Weisse Liste”) (35) which is a guidepost in the German health care system offering patients and their relatives support in their search for suitable doctors, hospitals, and nursing facilities. VICUR is an alternative checklist with healthcare background which does not include communication and patient safety aspects and was therefore introduced as a control condition to minimize Hawthorne effects.

A *satisfaction questionnaire* was designed on the basis of expert opinions to assess the potential influence of using checklists on the completeness, structure and duration of physician handovers. Also, the perceived influence on the patient outcome and the willingness to continue using the checklist were captured. Finally, the overall checklist was evaluated on the basis of grades (scale from 1 to 6, 1 being “excellent”, 6 being “very poor”) and free text evaluation.

### Outcome Measurements

For descriptive purposes, we assessed demographic data, specialization, professional experience in years, and professional status of the physicians. Next to physicians' data and handover duration, the patient outcome parameters SOFA score (for Days 1–5 after submission, with 2 days (48 h) being our primary outcome), mortality, length of stay (LOS), and reuptake on ICU were recorded during intervention using the IntelliSpace Critical Care and Anesthesia-system® (ICCA-system®), which is used by default in the ICUs of the University Hospital Aachen (UKA) to document patient data such as patient diagnoses, vital signs, medication and progress documentation, both by nursing staff and physicians. Communication with clinical IT systems and devices enabled precise information transfer (36). SOFA scores were recorded routinely each morning at ~06:00 A.M. These parameters had been considered important by a group of experts, who were interviewed using the Delphi method. As a quality indicator, handover duration was recorded automatically and invisibly for the physicians. Moreover, we evaluated compliance (i.e., the percentage of handovers for which the checklists were used) and satisfaction (grades from 1 to 6, 1 being “excellent,” 6 being “very poor”), via an online-based questionnaire.

### Sample Size

In concordance with the extensions of the CONSORT 2010 statement (37) for randomized pilot and feasibility trials, a formal sample size calculation is not required for pilot studies. Nevertheless, a sample size justification was conducted on the basis of the number of handovers being carried out regularly within a time period of 6 months. We estimated that about 1,700 patients would receive treatments on the seven ICU wards at the University Hospital Aachen during a 6-month period.

### Statistical Analysis

Statistical analyses were conducted using SPSS Statistics (Version 25; IBM Corp., Armonk, NJ, USA) and on the basis of the intention-to-treat principle. Analyses for patient-related endpoints were performed on cluster level. Mortality was defined as the ratio of deaths and the total number of patients (in %) during the respective period; reuptake was determined as the ratio of reuptakes and the total number of patients (in %) during the respective period. Unbiased estimates for treatment differences between these endpoints were calculated after Reed (38). First, mean SOFA scores were calculated for each of the seven wards for each period in both sequences. Second, for each ward, a *treatment contrast* C was calculated based on these mean values. For Arm 1, the calculation was based on the formula C1 = (2^*^A – B1 – B2); for Arm 2, the calculation was based on the formula C2 = (2^*^B – A1 – A2), respectively. The *treatment difference* was then calculated as the difference between treatment effects, i.e., C1 – C2. Significance testing was subsequently performed based on these ward-wise treatment differences. Handover durations were calculated on handover level and compared with independent samples t-tests between checklists and, for explorative purposes, with respect to weekday (working day, i.e., Monday–Friday vs. weekend, i.e., Saturday and Sunday) and daytime (morning shift change vs. evening shift change). Moreover, assessments of the checklists by the users were analyzed. Ratings of the checklists by the employees were aggregated over periods and compared between checklists by independent samples *t*-tests. Compliance was defined as the ratio of handovers using the checklists and the number of total possible handovers. Besides comparisons between checklists, we again investigated weekday and daytime effects on the frequency of checklist use. Differences in frequency distributions between checklists were assessed with χ^2^ tests. Significance for all statistical tests was assessed in a two-tailed fashion (if applicable). Significance levels were defined as *p* < 0.05 for all tests. All reported mean differences reflect the treatment difference (ISBAR_3_ – VICUR).

## Results

### Descriptive Statistics: Patient Data

From 1,882 patients on the wards in the respective time frame, 1038 met the inclusion criteria. 63.1% of the patients were male; mean patient age was 64.6 years. Patients were assigned from 15 different clinical departments, most frequently from the thoracic surgery, general surgery, and neurosurgery. Most of the admission diagnoses (*n* = 376) were diseases of the circulatory system. Thus, the total patient number exceeds our a-priori estimate (1,700 patients), and the final sample size of 1,038 patients meets the recommendations from the scientific literature (39–41).

### Descriptive Statistics: Physician's Data

Sixty-one physicians signed the written consent and participated. 60.7% of the physicians (*n* = 37) were residents, 18% were board certified specialists (*n* = 11), 19.7% (*n* = 12) were attending physicians and 1 function (1.6%) was not reported. The most frequently reported discipline was anesthesia with 48 (78.7%) physicians. 13.1% were surgeons and only four physicians (6.6%) belonged to internal disciplines.

### Clinical Outcome Parameters

#### SOFA Score

The results reported in Table 1 show a significant difference for the 1st 24 h time window after ICU admission, with higher SOFA scores for ISBAR_3_ compared to VICUR (*p* = 0.02) while the other time points yielded no significant differences. The primary outcome parameter was the SOFA score at the 2nd 24 h time window (48 h).

**Table 1:** Treatment difference (ISBAR_3_ – VICUR) in SOFA scores after ICU admission.

**SOFA score**	**Treatment difference (ISBAR_**3**_ – VICUR)**	***t***	***p***
First 24-h time window	2.19	3.35	0.02^*^
Second 24-h time window	1.78	1.48	0.20
Third 24-h time window	0.38	0.30	0.78
Fourth 24-h time window	1.16	0.64	0.60
Fifth 24-h time window	2.22	1.33	0.25

*SOFA, sepsis-related organ failure assessment; ICU, intensive care unit*;

* *significant at p < 0.05*.

#### Mortality, LOS, and Reuptake on ICU

Within 30 days after admission, the mortality rate of patients was 8.1%. No significant difference between the checklists emerged (mean difference 1.59, *t* = 0.30, and *p* = 0.77).

The mean LOS within 30 days after admission was 6.8 ± 8.8 days. No significant difference between the checklists emerged (mean difference 5.26, *t* = 1.47, and *p* = 0.20).

The reuptake rate of patients within 30 days after admission was 7.5%. No significant difference between the checklists emerged (mean difference −7.03, *t* = −1.43, and *p* = 0.21).

#### Handover Duration

Average handover duration was 66.32 ± 87.10 seconds for ISBAR_3_ and 43.91 ± 73.37 seconds for VICUR. Durations differed significantly between checklists, indicating a shorter duration for VICUR (*t* = 8.02, *p* > 0.001). No differences with respect to duration emerged between working days (Monday–Friday) and weekends (Saturday–Sunday) for both ISBAR_3_ (*t* = 0.20, *p* = 0.84) and VICUR (*t* = 1.02, *p* = 0.31). Remarkably, for VICUR, morning handovers were significantly shorter than evening handovers (*t* = 4.50, *p* < 0.001), whereas no such effect was observed for ISBAR_3_ (*t* = 0.75, *p* = 0.45).

#### Satisfaction and Compliance

Concerning satisfaction ISBAR_3_ achieved significantly better grades than VICUR (mean difference 0.87, *t* = 3.43, *p* < 0.001). Overall, compliance was 25.4% for ISBAR_3_ and 15.8% for VICUR. Thus, compliance was significantly higher for ISBAR_3_ (χ^2^ = 216.55, *p* < 0.001). 85.1% of all ISBAR_3_ handovers were on working days (i.e., 14.9% were on weekends), whereas the proportion was 77.2% on working days for VICUR handovers (i.e., 22.8% were on weekends). Assuming an equal distribution over all weekdays, we would expect 71.43% of handovers for working days and 28.57 of handovers for weekends. Thus, there was a culmination of checklist handovers on working days, which was more pronounced for the ISBAR_3_ condition. This difference in distributions between checklists was statistically significant (*X*^2^ = 33.54, *p* < 0.001). 58.2% of all ISBAR_3_ handovers took place in the morning shift change; 29.7% took place in the evening shift change. In other words, ISBAR_3_ was used almost twice as often in the morning as in the evening. For VICUR, 52.9% of handovers took place in the morning and 36.0% in the evening shift change (numbers missing to 100% were shift changes on other daytimes). Again, the difference in distributions between the two checklists was significant, confirming a relatively higher proportion for the morning shift change for ISBAR_3_ (*X*^2^ = 13.65, *p* < 0.001).

The satisfaction questionnaire was filled out by physicians 35 times (57.4%) in the first period, 31 times (50.8%) in the second period, and 27 times (44.3%) in the third period. The evaluation of the free text comments reflects the preference of ISBAR_3._ Overall, regardless of the checklist used, the criticism ranged from occurrence of technical problems over request for detailed description of the checklist items to request for extensive training. The contents of the VICUR checklist, in contrast to ISBAR_3_, were not considered to be relevant. Moreover, an increased expenditure of time as compared to the regular handover procedure was criticized for VICUR.

## Discussion

The aim of the present pilot study was to estimate effect sizes of a structured clinical handover checklist on patient-related objective outcome parameters and to investigate feasibility aspects with regard to an RCT on patient safety. Specifically, the study was the first RCT to define patient safety based on clinical outcome parameters. Results show the potential of an RCT to include large samples of patient handovers, but they also highlight a number of points to be considered. The randomized and controlled crossover design is a clear methodical strength of the study (42).

When comparing the checklists, results did not reveal a superiority concerning outcomes. In specific, no effect of the checklist was observed on our primary outcome parameter (SOFA score for the second 24 h time window/after 48 h). On the contrary, ISBAR_3_ even yielded higher average SOFA scores on a descriptive level. The treatment difference (ISBAR_3_ – VICUR) for this time point was 1.78, indicating a higher mean SOFA score for ISBAR_3_, although only on a descriptive and not on a statistically significant level. However, there is evidence that the latter does not argue against the use of ISBAR_3_ but may instead be attributed to different baseline levels in SOFA scores at the time of admission. In fact, the analysis of the SOFA scores yielded a difference between the checklists only for the first time point (first 24 h time window), which indicates differences already shortly after admission to the ICU. It appears likely that these differences were present already at the time of admission. In summary, the present data does not allow the estimation of an effect size for our primary outcome in favor of ISBAR_3_,, and we have no reason to assume that any effect in favor of VICUR (significant or not) can be attributed to the checklist; instead, a systematic difference in baseline levels is the likely explanation. Nonetheless, from the study findings there is no reason for us to believe that SOFA scores per se are not suited as outcome parameters for a future RCT on the effects of handover checklists.

The baseline difference in SOFA scores is remarkable, given the randomization and the crossover design. Although the scores were recorded during the regular daily visits and not at the time point of admission, it seems justifiable to assume that this difference was unrelated to the checklist in use and affected SOFA scores at all following time points. A possible explanation may be a random fluctuation of SOFA scores within as well as between the ICUs over time. Table 2 shows a considerable variation for the average day 1 SOFA scores over the three periods. Remarkably, this was most pronounced for the ICU Ward for Operative Intensive Care Medicine (OIM2) with the largest sample size,whereas the small weaning station (WEA) had relatively stable average values. This may result from different specializations and patient groups of the units. Patients on the WEA are generally already long-term treated and recovering, whereas post-abdominal-surgery patients on the OIM2 are usually in an acute condition. The latter may foster fluctuations in average illness severity, which is captured by SOFA scores. In addition, it should be considered that patients treated on the WEA have already been to an ICU and are in a clinically improved condition ready to be weaned from ventilation. This pre-selection may have caused the lower fluctuation on WEA.

**Table 2:** SOFA scores by ICU and sequence.

**ICU**	Sequence^*^	**Average SOFA score for first 24 h time window**		
		**(number of patients in parentheses)**		
		**Period 1**	**Period 2**	**Period 3**
OIM1	1	8.41 (36)	7.57 (64)	7.70 (60)
OIM2	2	8.00 (92)	9.21 (83)	7.66 (78)
OIM3	1	9.26 (60)	8.66 (65)	8.85 (61)
OIM4	2	7.58 (26)	8.92 (21)	8.92 (20)
OIM5	1	7.73 (48)	7.89 (65)	7.64 (58)
OIM6	1	6.76 (46)	6.54 (50)	6.86 (53)
WEA	2	6.89 (20)	7.27 (15)	7.58 (17)

SOFA, sepsis-related organ failure assessment; ICU, intensive care unit; OIM, operative intensive care medicine; WEA, weaning ward

* *1 = ISBAR_3_ – VICUR – VICUR; 2 = VICUR – ISBAR_3_ – ISBAR_3_; green = ISBAR_3_, blue = VICUR*.

Furthermore, as seen in our study, even randomized studies may be confronted with different baseline levels of clinical outcome parameters. In an ABB/BAA design, one sequence may be more affected by within-cluster fluctuations than the other if these fluctuations vary systematically between ICU types. Both effects are likely to be more pronounced in monocentric studies with a rather small number of clusters. However, for future studies we consider it essential to avoid such baseline confounds in patient outcomes, especially since sepsis scores also have a potential influence on other patient outcomes, such as mortality, reuptake, or duration of stay. To circumvent these methodical problems, we recommend the use of a stratified randomization according to specialization for future multicentre studies. To provide an adequate randomization in a single center design, we recommend a randomization on handover level instead of cluster level.

Besides the above-mentioned baseline differences, the missing effect of checklists on mortality rates may indicate that mortality per se is a too insensitive outcome parameter. Specifically, it may be necessary to consider that the death of a patient is rather frequent in an ICU (8.1% of all patients in our sample), whereas death due to a faulty handover is a rather rare event. In the vast majority of all cases, a faulty handover will have no negative consequences at all. Only those cases are critical where handover errors lead to treatment errors. A theory on how such treatment errors can occur and lead to death is explained in Reason's Swiss Cheese Model of System Accidents (43). According to Reason, accidents do not occur due to individual failures, but require a chain of failures caused by defects in various safety barriers. In addition, particular external circumstances must arise so that these defects emerge in a certain constellation entailing that the accident actually occurs. Thus, it can be assumed for handover processes that the potential failure during handover can only be detected if certain failures result in a certain constellation of treatment errors, occurring coincidently to external relevant circumstances (e.g., the increasing health condition of a patient caused by a hospital acquired infection making him or her more vulnerable) and end up in a fatal event. For further studies it thus seems reasonable to focus on deaths as a consequence of medical treatment errors. Additionally, larger sample sizes are required to detect differences in patient safety outcome parameters like mortality. This elaborate study has some limitations which are summarized in the following topics. An important point of discussion is that the study is conducted in a single-center design and further investigations are necessary to confirm a strong transferability. Furthermore, it is a challenge to define a good standard comparison group within the two options given. Comparing either the investigated intervention using digital handovers checklist with the current most representable situation in clinical departments, where handovers are done without a checklist is methodically very imprecise, or with content that does not concern patient safety issues difficult in terms of acceptance of the users thus challenging.

The randomized and controlled crossover design is a clear methodical strength of the study (42). However, looking at the satisfaction ratings our findings indicate that the two study arms (checklists) differed in their acceptance among the medical staff, suggesting that the perceived uselessness of VICUR was the major cause for the low compliance. VICUR was easily recognized as a control checklist by the physicians, which is also a likely explanation for the shorter duration of VICUR handovers. In a way, this corresponds to a kind of unvoluntary “self-unblinding” of the experimental condition. VICUR can thus be considered critical as a control condition. With regard to future multicentre studies, the use of control checklists such as VICUR should therefore be scrutinized. Instead, it appears more valid to compare a checklist to conventional handovers without a checklist. Here, potential Hawthorne effects could be avoided by informing the participants in both conditions that their performance is part of a study.

Furthermore, overall compliance was very low, with ISBAR_3_ being used in 25.4% and VICUR in only 15.8% of handovers. Under these circumstances clinical outcomes in both conditions are mainly based on handovers without any checklist and these low compliance rates drastically reduce statistical power. In order to increase the physicians' long-term motivation in future studies, incentives could be provided through an incentive system which analyses the physicians' needs and improvement suggestions such as simplifications of the daily workflow by integrating the checklist into the computer system and accessing it via tablet.

A remarkable finding was that, compared to VICUR, ISBAR_3_ was used preferably on working days and in the morning shift change. This is a very interesting aspect that, in our view, is a valuable puzzle piece for understanding the compliance with checklist use. A plausible explanation for this pattern of use is the presence of a senior physician during the handover/shift change in the morning on working days. In particular, we assume that senior physicians foster compliance by encouraging the use of a handover checklist since, based on their knowledge and experience, they consider it useful. Remarkably, this effect is mainly limited to ISBAR_3_, which is again in line with the perceived uselessness of VICUR.

There are two tentative conclusions that can be drawn from these findings. First, compliance with checklist use may benefit from an education on background and purpose of checklists. Second, control checklists such as VICUR are seemingly inadequate control conditions. Based on the present findings, it should even be taken into consideration that their use in studies may be harmful. If they are perceived as useless and if their use is not encouraged by senior physicians, it appears conceivable that they evoke the general impression of checklists being a waste of time. Especially for young residents, this would be a highly undesired effect.

In addition, compliance and the overall outcome could be increased by training in handover practices (15), to improve and fasten handover during stressful situations (31). Indeed, findings on the WHO Surgical Safety Checklist suggest that the effect of a checklist on patient safety aspects may be substantially larger when combined with team training on its correct application (44).

As these are complex strains, more experience has to be gathered and analyzed in further investigations. The authors are convinced that the present study provides an important scientific contribution to this topic and serves as guidance for further research.

## Conclusion

Medical handovers are a burning issue in medicine concerning patient safety. Their continuous application and improvement are important goals. The present pilot study illustrates the complexity of this topic and shows both the potential and the pitfalls concerning outcome parameters and feasibility that should be considered in a future multicentre study. Further research is needed to measure the direct impact of structured handovers on patient outcomes with unambiguous parameters.

## Data Availability Statement

The raw data supporting the conclusions of this article will be made available by the authors, without undue reservation.

## Ethics Statement

The study was reviewed and approved by the Ethics Committee of the Faculty of Medicine of RWTH Aachen University (EK 075/17) on 24 May 2017. The study protocol was designed and performed in line with the Declaration of Helsinki. Written informed consent was obtained from all physicians working on ICU wards during data acquisition. They were informed that their participation was voluntary and could be discontinued at any time without explanation or any disadvantages.

## Author Contributions

NV contributed substantially to the acquisition, analysis, and interpretation of the data and has written the manuscript. SS, SB, MS, and GM contributed substantially to the interpretation of the data and have critically revised the manuscript for important intellectual content. SS, MK, and LV contributed substantially to the designing of this study, the statistical analysis, and interpretation of the data. SS made substantial contributions to the planning and designing the study protocol, analysis, and interpretation of the data, assisted to the writing of the manuscript as well he revised it critically for important intellectual content. SS supervised the study and supported NV as senior investigator. All authors reviewed and revised the manuscript. All authors have made contributions to the manuscript.

## Conflict of Interest

The authors declare that the research was conducted in the absence of any commercial or financial relationships that could be construed as a potential conflict of interest.

## Abbreviations

AHRQ: Agency for Healthcare Research and Quality
DGAI: German Society of Anaesthesiology and Intensive Care Medicine
ICAA-System: IntelliSpace Critical Care and Anesthesia-System
ICU: Intensive care unit
IHI: Institute for Healthcare Improvement
ISBAR_3_, Identity, Situation, Background, Assessment, Recommendation, Read-back: Risk
LOS: Length of stay
OIM: ICU Ward for Operative Intensive Care Medicine
RCT: Randomized controlled trial
SOFA: Sepsis-related organ failure assessment
UKA: University Hospital Aachen
VICUR, Vaccination status, Insurance status, Contact person, Utilization: Rehabilitation
WEA: Weaning station
WHO: World Health Organization.

## References

[1] World Health Organization (WHO). Patient Safety. WHO (2020). Available online at: http://www.who.int/patientsafety/en/ (accessed July 30, 2020).

[2] World Health Organization (WHO). Data and Statistics. (2020). Available online at: https://www.euro.who.int/en/health-topics/Health-systems/patient-safety/data-and-statistics (accessed July 30, 2020).

[3] De Vries EN Ramrattan MA Smorenburg SM Gouma DJ Boermeester MA. The incidence and nature of in-hospital adverse events: a systematic review. BMJ Qual Saf . (2008) 17:216–23. 10.1136/qshc.2007.023622 18519629 PMC2569153

[4] Brennan TA Gawande A Thomas E Studdert D. Accidental deaths, saved lives, and improved quality. N Engl J Med. (2005) 353:1405–9. 10.1056/NEJMsb051157 16192489

[5] Eisold C Heller AR. Risikomanagement in anästhesie und intensivmedizin. Anaesthesist. (2016) 65:473–88. 10.1007/s00101-016-0189-9 27273109

[6] Jürgens JJS. Das Kommunikationsmodell SBAR - Eine systematische Literaturrecherche zur Effektivität des strukturierten Kommunikationsmodells SBAR in Bezug auf Patientensicherheit. Dissertation, Universität zu Köln (2016).

[7] Flemming D Hübner U. How to improve change of shift handovers and collaborative grounding and what role does the electronic patient record system play? Results of a systematic literature review. Int J Med Inform. (2013) 82:580–92. 10.1016/j.ijmedinf.2013.03.004 23628146

[8] Mukherjee S. A precarious exchange. N Engl J Med. (2004) 351:1822–4. 10.1056/NEJMp048085 15509813

[9] Okie S. An elusive balance – residents' work hours and the continuity of care. N Engl J Med. (2007) 356:2665–7. 10.1056/NEJMp078085 17596598

[10] Kitch BT Cooper JB Zapol WM Marder JE Karson A Hutter M. Handoffs causing patient harm: a survey of medical and surgical house staff. Jt Comm J Qual Patient Saf . (2008) 34:563–70. 10.1016/S1553-7250(08)34071-9 18947116

[11] Molesworth BRC Estival D. Miscommunication in general aviation: the influence of external factors on communication errors. Saf Sci. (2015) 73:73–9. 10.1016/j.ssci.2014.11.004

[12] The Joint Commission. Annual Report: Improving America's Hospitals. (2007). Available online at: https://www.jointcommission.org/-/media/tjc/documents/accred-and-cert/hap/annual-report/2007_annual_reportpdf.pdf?db=web&hash=2092D322E8296BCE340B5FF331BDAD82 (accessed July 30, 2020).

[13] Eggins S Slade D. Communication in clinical handover: improving the safety and quality of the patient experience. J Public Health Res. (2015) 4:666. 10.4081/jphr.2015.666 26753165 PMC4693345

[14] McSweeney ME Lightdale JR Vinci RJ Moses J. Patient handoffs: pediatric resident experiences and lessons learned. Clin Pediatr (Phila). (2011) 50:57–63. 10.1177/0009922810379906 20837612

[15] Starmer AJ Sectish TC Simon DW Keohane C McSweeney ME Chung EY. Rates of medical errors and preventable adverse events among hospitalized children following implementation of a resident handoff bundle. JAMA. (2013) 310:2262. 10.1001/jama.2013.281961 24302089

[16] Stewart KR. SBAR, Communication, and Patient Safety: An Integrated Literature Review. Dissertation, University of Tennessee at Chattanooga (2016).

[17] Beuken JA Verstegen DML Dolmans DHJM Van Kersbergen L Losfeld X Sopka S. Going the extra mile - cross-border patient handover in a European border region: qualitative study of healthcare professionals' perspectives. BMJ Qual Saf. (2020) 29:980–7. 10.1136/bmjqs-2019-010509 32132145

[18] Robertson ER Morgan L Bird S Catchpole K McCulloch P. Interventions employed to improve intrahospital handover: a systematic review. BMJ Qual Saf . (2014) 23:600–7. 10.1136/bmjqs-2013-002309 24811239

[19] Hoffmann B Rohe J. Patient safety and error management. Dtsch Aerzteblatt Online. (2010) 107:92–100. 10.3238/arztebl.2010.0092 PMC2832110 20204120

[20] Starmer AJ Spector ND Srivastava R West DC Rosenbluth G Allen AD. Supplementary appendix to changes in medical errors after implementation of a handoff program. N Engl J Med. (2014) 371:1803–12. 10.1056/NEJMsa1405556 25372088

[21] de Vries EN Prins HA Crolla RMPH den Outer AJ van Andel G van Helden SH. Effect of a comprehensive surgical safety system on patient outcomes. N Engl J Med. (2010) 363:1928–37. 10.1056/NEJMsa0911535 21067384

[22] McCarney R Warner J Iliffe S van Haselen R Griffin M Fisher P. The Hawthorne Effect: a randomised, controlled trial. BMC Med Res Methodol. (2007) 7:30. 10.1186/1471-2288-7-30 17608932 PMC1936999

[23] Lancaster GA Dodd S Williamson PR. Design and analysis of pilot studies: recommendations for good practice. J Eval Clin Pract. (2004) 10:307–12. 10.1111/j.2002.384.doc.x 15189396

[24] Uschner D Schindler D Heussen N Hilgers R-D. randomizeR: an R package for the assessment and implementation of randomization in clinical trials. J Stat Softw. (2018) 85:1–22. 10.18637/jss.v085.i0830505247

[25] Randmaa M Swenne CL Mårtensson G Högberg H Engström M. Implementing situation-background-assessment-recommendation in an anaesthetic clinic and subsequent information retention among receivers. Eur J Anaesthesiol. (2016) 33:172–8. 10.1097/EJA.0000000000000335 26760400

[26] Holtel M Piwernetz K Pilz S Poimann H Rode S Stapenhorst K Weber H. Alles Gesagt - Alles Verstanden? Eine Klinik, Eine Sprache: Sicher Kommunizieren Mit SBAR. f&w (2016). Available online at: https://www.bibliomedmanager.de/fw/artikel/17130-eine-klinik-eine-sprache (accessed August 8, 2020).

[27] Randmaa M Mårtensson G Leo Swenne C Engström M. SBAR improves communication and safety climate and decreases incident reports due to communication errors in an anaesthetic clinic: a prospective intervention study. BMJ Open. (2014) 4:e004268. 10.1136/bmjopen-2013-004268 24448849 PMC3902348

[28] Müller M Jürgens J Redaèlli M Klingberg K Hautz WE Stock S. Impact of the communication and patient hand-off tool SBAR on patient safety: a systematic review. BMJ Open. (2018) 8:e022202. 10.1136/bmjopen-2018-022202 30139905 PMC6112409

[29] World Health Organization (WHO). WHO-Kollaborationszentrum für Lösungskonzepte zur Patientensicherheit. (2007). Available online at: https://www.jointcommissioninternational.org/-/media/deprecated-unorganized/imported-assets/jci/assets/jci-patient-safety-solutions/patientsolutionsgermanpdf.pdf?db=web&hash=4134231E235CDD29A16125E167B9AF60 (accessed July 30, 2020).

[30] Schyve PM Brockway M Carr M Giuntoli A Mcneily M Reis P. The Joint Commission Guide to Improving Staff Communication, 2nd edn. Oakbrook Terrace, IL: Joint Commission on Accreditation of Healthcare Organizations (2009).

[31] von Dossow V Zwissler B. Recommendations of the German Association of Anesthesiology and Intensive Care Medicine (DGAI) on structured patient handover in the perioperative setting. Anaesthesist. (2016) 65:1–4. 10.1007/s00101-016-0237-5 27900413

[32] Institute for Healthcare Improvement. SBAR Tool: Situation-Background-Assessment-Recommendation. (2020). Available online at: http://www.ihi.org/resources/Pages/Tools/SBARToolkit.aspx (accessed July 30, 2020).

[33] United States. Department of Defense, United States. Agency for Healthcare Research and Quality, United States. Office of the Assistant Secretary of Defense (Health Affairs). TRICARE Management Activity. TeamSTEPPS^®^: Strategies and Tools to Enhance Performance and Patient Safety. Pocket Guide. TRICARE. (2010). 35 p.

[34] Greenstein EA Arora VM Staisiunas PG Banerjee SS Farnan JM. Characterising physician listening behaviour during hospitalist handoffs using the HEAR checklist. BMJ Qual Saf . (2013) 22:203–9. 10.1136/bmjqs-2012-001138 23258389 PMC4375540

[35] Krankenhaus-Checkliste | Weisse Liste. Available online at: https://www.weisse-liste.de/export/sites/weisseliste/de/.content/pdf/informationen/Checkliste_fuer_den_Krankenhausaufenthalt.pdf (accessed July 30, 2020).

[36] IntelliSpace Critical Care and Anesthesia. Philips Healthcare. Available online at: https://www.philips.at/healthcare/product/HCNOCTN332/intellispace-critical-care-and-anesthesia (accessed July 30, 2020).

[37] Eldridge SM Chan CL Campbell MJ Bond CM Hopewell S Thabane L. CONSORT 2010 statement: extension to randomised pilot and feasibility trials. Pilot Feasibility Stud. (2016) 2:64. 10.1186/s40814-016-0105-8 27965879 PMC5154046

[38] Reed JF. Extension of Grizzle's classic crossover design. J Mod Appl Stat Methods. (2011) 10:322–8. 10.22237/jmasm/1304224080

[39] Julious SA. Sample size of 12 per group rule of thumb for a pilot study. Pharm Stat. (2005) 4:287–91. 10.1002/pst.185

[40] Cocks K Torgerson DJ. Sample size calculations for pilot randomized trials: a confidence interval approach. J Clin Epidemiol. (2013) 66:197–201. 10.1016/j.jclinepi.2012.09.002 23195919

[41] Browne RH. On the use of a pilot sample for sample size determination. Stat Med. (1995) 14:1933–40. 10.1002/sim.4780141709 8532986

[43] Effective Public Health Praxtice Project. Quality Assessment Tool for Quanitative Studies. (2003). Available online at: https://merst.ca/wp-content/uploads/2018/02/quality-assessment-tool_2010.pdf (accessed July 30, 2020).

[44] Reason J. Managing the Risks of Organizational Accidents. Burlington: Ashgate Publishing Company (1997). p. 252.

[45] Urbach DR Govindarajan A Saskin R Wilton AS Baxter NN. Introduction of Surgical Safety Checklists in Ontario, Canada. N Engl J Med. (2014) 370:1029–38. 10.1056/NEJMsa1308261 24620866

